# Jiedu-Yizhi Formula Improves Cognitive Impairment in an A*β*_25–35_-Induced Rat Model of Alzheimer's Disease by Inhibiting Pyroptosis

**DOI:** 10.1155/2022/6091671

**Published:** 2022-03-16

**Authors:** Jiale Wang, Xiaoting Zhu, Yuhui Li, Pengqi Zhang, Tianye Wang, Mingquan Li

**Affiliations:** ^1^School of Integrated Chinese and Western Medicine, Changchun University of Chinese Medicine, Changchun 130117, Jilin, China; ^2^School of Chinese Medicine, Changchun University of Chinese Medicine, Changchun 130117, Jilin, China

## Abstract

Jiedu-Yizhi formula (JDYZF) is prescribed for the treatment of Alzheimer's disease (AD) and was created by Jixue Ren, a master of traditional Chinese medicine, based on the “marrow deficiency and toxin damage” theory. In our clinic, this formula has been used for the treatment of AD for many years and has achieved good results. However, the mechanism by which JDYZF improves cognitive impairment has not been determined. In this study, we confirmed that orally administered JDYZF reversed the cognitive deficits in an A*β*_25–35_-induced rat model, increased the number of neurons in the hippocampal CA1 area, improved their structure, decreased the deposition of *β*-amyloid (A*β*), reduced the expression of proteins related to the NLRP3/Caspase-1/GSDMD and LPS/Caspase-11/GSDMD pyroptosis pathways, and reduced the levels of interleukin 1*β* (IL-1*β*) and IL-18, thereby inhibiting the inflammatory response. In addition, JDYZF exerted no hepatotoxicity in rats. In short, these results provide scientific support for the clinical use of JDYZF to improve the cognitive function of patients with AD.

## 1. Introduction

Alzheimer's disease (AD) is characterized by the degeneration of the central nervous system. AD is the most common cause of dementia, and its symptoms include memory loss, personality changes, and social and emotional problems [1]. At present, approximately 50 million patients are suffering from AD worldwide. This number continues to rapidly increase and is expected to reach 152 million by 2050 [2]. Owing to its hidden onset, difficult diagnosis, and lack of a cure, AD imposes substantial economic and medical burdens on individuals and society [3, 4].

Many hypotheses have been proposed regarding the aetiological and pathological mechanisms of AD, but no exact theory has been confirmed. At present, drugs such as donepezil and memantine improve the symptoms of AD for only a short time. However, these agents cannot reverse or delay the progression of AD and have some side effects and toxicity [5]. New drugs that target the typical pathological characteristics of AD—senile plaques (SPs) formed by *β*-amyloid (A*β*) and neurofibrillary tangles (NFTs) caused by hyperphosphorylation of the tau protein—have achieved favourable results in only animals and not in clinical experiments [6, 7]. Therefore, under the guidance of alternative and integrated medicine, it is particularly important to select known effective methods for the treatment of AD, such as acupuncture, electroacupuncture, music therapy, aromatherapy, pulsed electromagnetic fields, and traditional Chinese medicines, to improve the clinical symptoms to the greatest extent and explore the mechanisms of these treatment methods [8–13]. Among them, traditional Chinese medicine formulas with multitarget therapeutic effects have been widely reported to improve the cognitive function of AD patients and have gradually attracted the attention of researchers [14].

Jiedu-Yizhi formula (JDYZF) is a traditional Chinese medicine compound that tonifies the kidney and marrow, resolves phlegm, and activates blood circulation and detoxification processes that were developed by Ren Jixue, a master of Chinese medicine. Our clinical department has used this formula for many years, and this treatment has a significant effect on AD. In a previous study on the mechanism of JDYZF, we found that the extract of *Coptis chinensis* polysaccharide, the main drug component of JDYZF, improved the lifespan of the transgenic *Caenorhabditis elegans* CL4176 model of AD, reduced the paralytic rate, inhibited nematode head A*β* deposition, increased heat shock protein hsp16.2 and hsp16.41 gene expression levels [15], reduced the A*β*_25–35_-induced expression of cleaved-caspase-3 and Bax in PC12 cells, increased the expression of Bcl-2 and inhibited apoptosis [16]. However, the mechanism of JDYZF in the treatment of AD remains unclear.

A strong inflammatory reaction occurs in the AD brain, and A*β* can activate microglia to trigger neuroinflammation; thus, neuroinflammation may play an important role in the pathogenesis of AD [17]. In recent years, pyroptosis has been shown to potentially promote neuroinflammation. Pyroptosis is a highly inflammatory programmed cell death mechanism mediated by cysteinyl aspartate-specific proteinases (caspases). Its characteristic manifestations are swelling of the cell body, rupture of the cell membrane, and release of proinflammatory factors to trigger an inflammatory cascade reaction [18, 19]. Pyroptosis is achieved through the assembly of inflammasomes, which are pattern recognition receptors such as NOD-like receptors (NLRs, including NLRP1 and NLRP3) that recognize pathogen-associated molecular patterns (PAMPs) and endogenous damage-associated molecular patterns (DAMPs) that recruit the adaptor protein apoptosis-associated speck-like protein containing a CARD (ASC). This adaptor is further connected to the downstream receptor caspase-1. The aggregation of caspase-1 leads to its autocleavage and to the production of its active form, which can induce the maturation of proinflammatory cytokines such as interleukin 1*β* (IL-1*β*) and IL-18. Concomitantly, gasdermin D (GSDMD) is cleaved to produce an *N*-terminal fragment (GSDMD-NT) that can initiate pyroptosis. GSDMD-NT can perforate the cell membrane, leading to cell death and to the outflow of proinflammatory factors [20–23]. Among the NLR family members, NLRP3 is the most well studied. NLRP3 is expressed by neuronal cells and microglia and can be activated by A*β*- and A*β*-induced oxidative stress products such as reactive oxygen species (ROS) and other substances to initiate pyroptosis [24, 25]. In addition, lipopolysaccharides (LPSs) in the cytosol can directly induce the activation of caspase-4/caspase-5 in humans or caspase-11 in rodents and the cleavage of GSDMD to cause nerve cell pyroptosis [26]. Therefore, altering pyroptosis and inhibiting neuritis could be breakthroughs in the treatment of AD. At the same time, inflammation is closely related to blood stasis [27]. Whether JDYZF, which ameliorates blood stasis, exerts a therapeutic effect by altering pyroptosis and inhibiting inflammatory reactions remains unknown.

In this study, we evaluated the effects of JDYZF on the cognitive and memory function of AD model rats. We also assessed the effects of JDYZF on the expression of A*β* and the NLRP3/caspase-1/GSDMD and LPS/caspase-11/GSDMD pyroptosis pathways to provide scientific support for its clinical application.

## 2. Materials and Methods

### 2.1. Animals

Adult male Sprague–Dawley rats (weight: 200–220 g) were purchased from Changchun Yisi Experimental Animal Technology Co., Ltd. (Changchun, China). According to the regulations of the experimental animal centre of the Changchun University of Chinese Medicine, the rats were allowed to drink freely and were housed in a feeding room at a temperature of 25 ± 3°C and a relative humidity of 55 ± 5% on a 12-hour light/dark cycle. The animal experimental procedure was approved by the Experimental Animal Ethics Committee of the Changchun University of Chinese Medicine (no. 2021207).

### 2.2. Preparation of the A*β*_25-35_ Oligomer

One milligram of A*β*_25-35_ dry powder (A4559, Sigma) was dissolved in 500 *μ*L of 0.9% normal saline to make a 2 *μ*g/*μ*L solution; the solution was sonicated for 5 minutes using a bath sonicator and then incubated at 37°C for 7 days. The incubated A*β*_25–35_ became flocculent and was stored in a refrigerator at 4°C for later use [28, 29].

### 2.3. Preparation of the JDYZF Decoction

JDYZ is composed of seven Chinese medicines, *Coptis*, *wine-treated rhubarb*, *Ligusticum wallichii*, *Pheretima*, *tortoise shell glue*, *Cornus officinalis*, and *Alpiniae oxyphyllae fructus*. The traditional Chinese medicines were purchased from Hongjian Pharmacy (Changchun, China). These herbs were mixed at a ratio of 1:1:1:1:1:1:2, soaked in distilled water (in a volume 5 times the volume of the medicine) for 1 hour, boiled for 1 hour, and then boiled twice. The extraction solutions were combined and concentrated to 1.0 g/mL, placed in sterile containers, sealed, and stored at −20°C.

### 2.4. Animal Modelling and Treatment

SD rats were anaesthetized with sodium pentobarbital and fixed on a brain stereotactic instrument. Holes were drilled in the rat skull 3 mm below the anterior fontanelle and 2 mm on both sides of the midline. After detecting the dura mater with a microsyringe, the needle was lowered 2.6 mm into the hippocampal CA1 area. Five microlitres of the A*β*_25-35_ solution was injected within 5 minutes. After 15 minutes, the needle was slowly withdrawn over a period of 5 minutes. The wound was quickly closed with paraffin after the ejection of the needle [30]. Seven days after the injection, the rats were randomly divided into five groups. The rats were administered an equivalent dose by gavage based on the body surface areas of humans and rats. Eleven rats each in the low-dose group (JDYZ.L), middle-dose group (JDYZ.M), and high-dose group (JDYZ.H) received 3.6 g/kg, 7.2 g/kg, and 14.4 g/kg JDYZF decoction, respectively, by gavage. Meanwhile, 10 rats in the positive drug group (PG) were administered donepezil hydrochloride (0.9 mg/kg) by gavage. Furthermore, 10 rats in the model group (MG) and 9 rats in the control group (CG) were administered normal saline (1 mL/100 g) by gavage. All animals were treated once per day for 8 weeks.

### 2.5. Morris Water Maze Test

The Morris water maze test was performed after 8 weeks of drug intervention. The water maze (diameter × height: 150 cm × 50 cm) was equally divided into four quadrants. Different patterns were placed on the wall of each quadrant to serve as clues. The platform (diameter: 10 cm) was placed 1 cm below the water surface of the fixed quadrant. The water was dyed black with melanin to ensure that the rats could not see the platform. A total of four water entry points were placed in each quadrant. The water temperature was maintained between 23°C and 27°C. Each rat was placed at the entry point facing the pool wall and was allowed 60 s to find and climb the platform. The time it took to find and climb the platform was recorded as the escape latency. If the rat did not climb the platform within the specified time, the time was recorded as 60 s. Subsequently, the rats were allowed to stay on the platform for 15 s, with an interval of 5 minutes between each entry. The experiment was performed for 4 days, and the order of entry points differed each day. Four days later, the platform was removed, and the rats were placed in the quadrant opposite the platform for the probe trial. Overall, the following information was recorded: the time it took for them to swim in the platform quadrant within 60 s, and the time it took for them to cross the platform [31]. The EthoVision XT, Version 11.0 system (Noldus, Netherlands) was used for recording and analysis.

### 2.6. Haematoxylin and Eosin (HE) Staining

The anaesthetized rats were injected intraperitoneally with 2% pentobarbital sodium (45 mg/kg) 24 hours after the water maze test. The required organs were collected quickly and stored according to the regulations. The hippocampus and liver were embedded after dehydration, cut into 5-µm-thick sections, and stained with HE [32]. The pathological changes in the hippocampus and liver were observed under an optical microscope.

### 2.7. Western Blotting

The rat hippocampi were homogenized and placed in a radioimmunoprecipitation assay lysis buffer containing protease and phosphatase inhibitors and phenylmethylsulfonyl fluoride. The samples were lysed on ice for 30 minutes and centrifuged at 13,000 r/min for 10 minutes at 4°C, and the supernatant was aspirated. The bicinchoninic acid assay was performed to determine the protein concentration. The samples were then stored at −80°C until further use. Western blotting was performed using standard protocols [33]. The membranes were incubated overnight with primary antibodies, including anti-NLRP3 (1:500, Novus, USA), anti-GSDMD (1:1000, Abcam, UK), anti-caspase-1 (1:1000, Novus, USA), and anti-caspase-11 (1:200, Novus, USA), and then with the appropriate secondary antibodies. ImageJ software was used to measure the gray values of the target bands.

### 2.8. Immunohistochemistry

The hippocampal slices were prepared at a thickness of 4 *μ*m and then incubated with 0.3% hydrogen peroxide (which was added dropwise) at room temperature for 10 minutes to block endogenous peroxidase activity. The slices were washed 3 times with phosphate-buffered saline (PBS) and blocked with 5% bovine serum albumin for 30 minutes. The slices were incubated overnight with A*β* (MOAB-2, recognizes unaggregated, oligomeric and fibrillar forms of beta amyloid 42 and unaggregated beta amyloid 40, 1:500, Novus, USA), NLRP3 (1:50, Novus, USA), and GSDMD (1:1000, Abcam, UK) antibodies. Subsequently, the slices were washed 3 times with PBS and incubated with biotin secondary antibodies for 20 minutes and then treated with 3,3′-diaminobenzidine for 3 to 5 minutes. After washing, they were counterstained with haematoxylin and mounted after dehydration [34]. The slices were observed using a Cytation 5 (BioTek, USA) image reader. ImageJ software was used to analyse the average optical density (AOD) values and positive cell counts.

### 2.9. Enzyme-Linked Immunosorbent Assay (ELISA)

A*β*_1-42_ (Elabscience, China), IL-1*β* (mlbio, China), and IL-18 (mlbio, China) ELISA kits were used to determine the levels of A*β*_1-42_ and the inflammatory factors IL-1*β* and IL-18 in the hippocampus, cortex, colon, and serum. A microplate reader was used to detect the absorbance of the sample at 450 nm.

### 2.10. Statistical Analysis

All data are presented as the mean ± standard deviation. SPSS 20 software was used for data analysis. One-way analysis of variance (one-way ANOVA) was used to compare the differences among multiple groups. If the variances were uniform, the least significant difference test was used. If the variances were not uniform, Dunnett's T3 test was used. A *p* value of <0.05 was considered statistically significant, and a *p* value of <0.01 was considered highly statistically significant.

## 3. Results

### 3.1. JDYZF Can Improve Cognitive Impairment in AD Rats Induced with A*β*_25–35_

**Figure 1:**
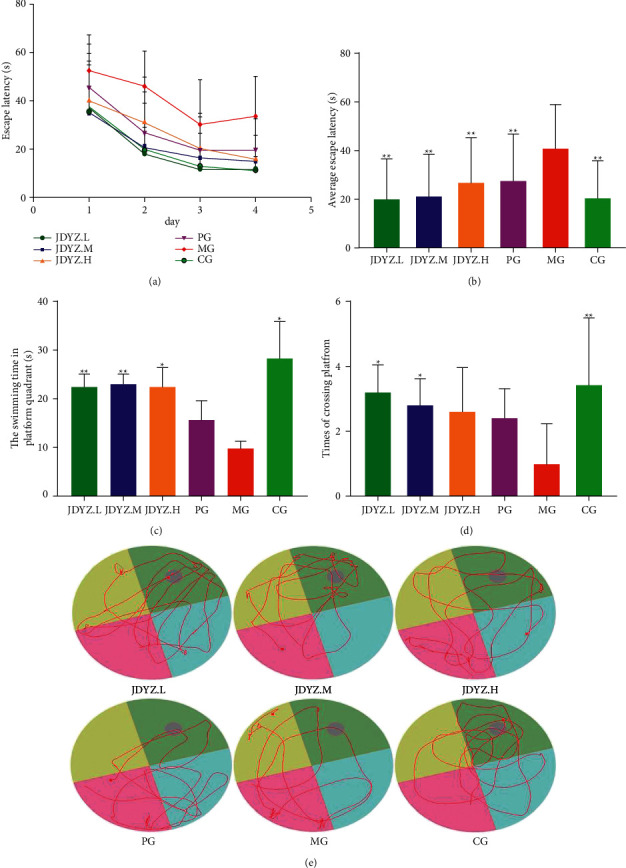
Oral administration of JDYZF rescues cognitive deficits in A*β*_25–35_-induced rats: (a) the rats in each group were trained in the water maze for 4 days, and the escape latency (expressed as means ± SEM) was gradually decreasing. The average escape latency (b), the swimming time in platform quadrant (c), and the number of crosses in the quadrant (d) were measured and recorded. (e) Representative swimming trajectories of different groups rats after removing the platform. Use one-way ANOVA to compare differences between multiple groups. ^*∗*^*p* < 0.05 and ^*∗∗*^*p* < 0.01, other groups compared with the model group.

The spatial learning and memory of rats were evaluated using the water maze test by calculating the escape latency of each group during the first 4 days and the average escape latency. The results showed no significant difference between the MG and other groups (except JDYZ.M) on the first day. On days 2 to 4, the escape latencies of the MG and other groups differed significantly (Figure 1(a)). The average escape latency was significantly higher in the MG group than in the other groups (*p* < 0.01) (Figure 1(b)). In addition, after the removal of the platform, the rats in the MG group spent less time swimming in the quadrant in which the platform was located (*p* < 0.05 except PG) (Figure 1(c)) and crossed the platform fewer times than those in the other groups (*p* < 0.05 except PG and JDYZ.H) (Figure 1(d)). Moreover, after removing the platform, the swimming trajectories of the MG and PG groups were sparse and irregular, while that of the CG group was dense and tended to be in the quadrant with the platform; the trajectories of the JDYZF groups were similar to those of the CG group to varying degrees (Figure 1(e)). This finding indicates that the injection of A*β*_25–35_ into the hippocampal CA1 area impaired the spatial learning and memory abilities of the rats. JDYZF and donepezil attenuated the learning and memory impairments to varying degrees. Notably, the effect of JDYZF was more prominent, especially in the JDYZ.L group.

### 3.2. JDYZF Attenuates Hippocampal Neuron Damage in AD Rats Induced with A*β*_25–35_

**Figure 2:**
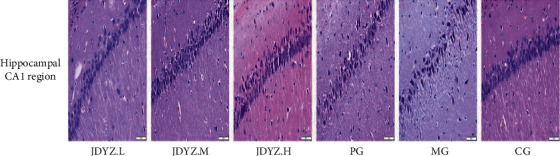
Representative histopathological photos of hippocampal CA1 area tissue sections from each group. JDYZF reduces A*β*_1–42_ levels in multiple tissues in AD model rats.

The deposition of A*β* can lead to neuronal cell loss and degeneration, which are the basis for the impairment of cognitive function in AD [35]. H&E staining showed that the pyramidal cells in the hippocampal CA1 area in the CG had the following features: tightly arranged, distinct layers, clear and complete morphology, rich cytoplasm, round nuclei, obvious nucleoli, and normal staining. In addition, the MG had the following features: disordered pyramidal cell arrangement, reduced number of layers, changed morphology, unclear boundary between the cytoplasm and nucleus, pyknotic nuclei, and deepened staining. The number and morphological structure of pyramidal cells in the CA1 area of the hippocampus were greatly recovered in the JDYZ.M, JDYZ.H, and PG groups compared with the MG. However, the effect was not as good as that in the JDYZ.L group (Figure 2). This finding indicates that JDYZF has some protective effect on hippocampal neurons.

**Figure 3:**
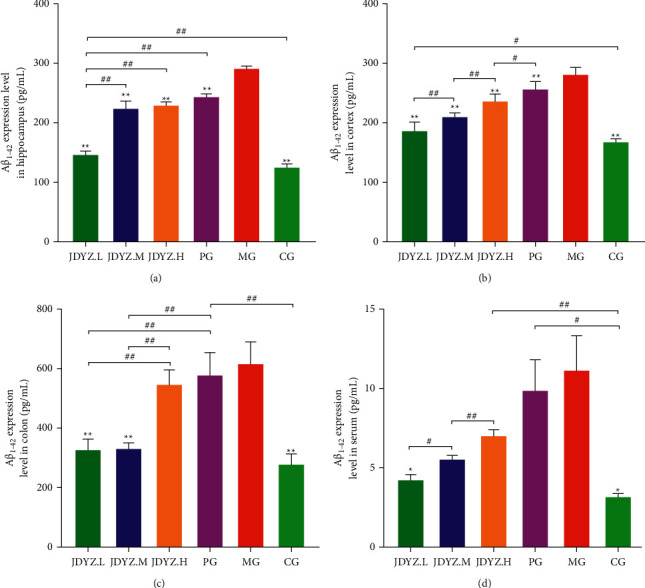
JDYZF oral administration can reduce the content of A*β*_1–42_ in the hippocampus (a), cerebral cortex (b), colon (c), and serum (d) of AD rats induced by A*β*_25–35_. ^*∗*^*p* < 0.05 and ^*∗∗*^*p* < 0.01, other groups compared with the model group. In addition to the model group, ^*#*^*p* < 0.05 and ^*##*^*p* < 0.01, and the other groups are compared in pairs.

The A*β*_23–35_ fragment is neurotoxic to A*β*_1–42_; after brain injection, the A*β*_23–35_ fragment causes a large amount of expression and deposition of A*β* oligomers, thereby triggering AD [36]. Owing to the presence of the gut-brain axis, AD cause changes in the intestinal ecology. Intestinal bacteria may increase the secretion of A*β* homologous proteins in the intestine, spread through the gut-brain axis to the brain, and trigger a vicious cycle of A*β* deposition [1]. We used an ELISA kit to measure the expression levels of A*β*_1–42_ in the hippocampus, cortex, colon, and serum samples of rats in each group. Compared with the vehicle injection, the A*β*_23–35_ hippocampal injection increased the expression level of A*β*_1–42_ in the rat hippocampus, cortex, colon, and serum. Orally administered donepezil slightly attenuated the increases in the A*β*_1–42_ levels; however, the effect was not as good as that of the JDYZF concoctions, especially that of JDYZ.L (Figures 3(a)–3(d)). This finding indicates that JDYZF can reduce the expression level of A*β* in AD model rats.

### 3.3. JDYZF Inhibits the Expression of Proteins Related to the Pyroptosis Pathway

**Figure 4:**
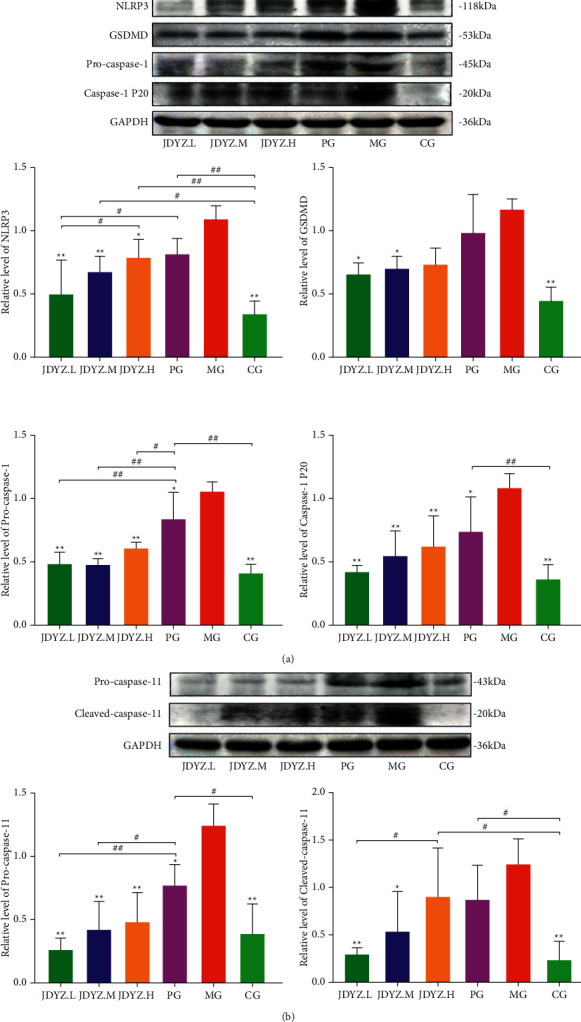
Compared to MG, oral administration of JDYZF and donepezil can reduce the expression levels of NLRP3, GSDMD, pro-caspase-1, and caspase-1 p20 in AD rats hippocampi (a). In addition, by reducing the expression of caspase-11 precursor and spliceosome, the activity of the nonclassical pyrolysis pathway is inhibited and the cognitive impairment of AD rats is improved (b). The relative level is equal to the gray value of the relevant protein divided by the gray value of GAPDH. ^*∗*^*p* < 0.05 and ^*∗∗*^*p* < 0.01, other groups compared with the model group. ^*#*^*p* < 0.05 and ^*##*^*p* < 0.01, the other groups are compared in pairs except the model group.

**Figure 5:**
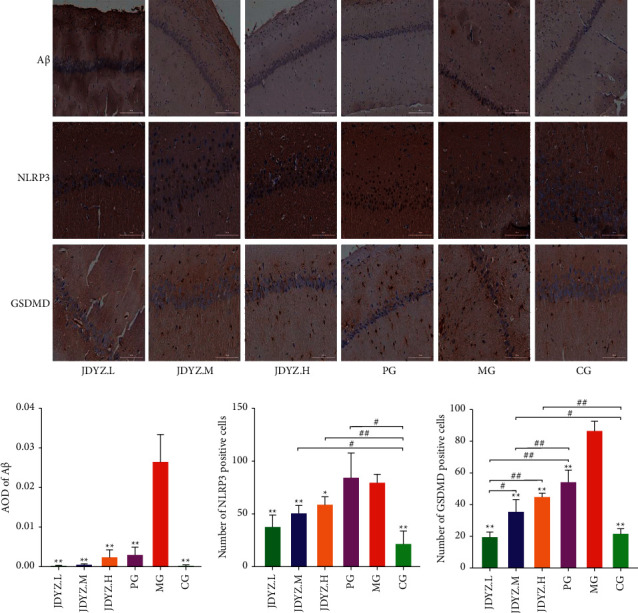
Oral administration of JDYZF can reduce the deposition of A*β* and the expression of NLRP3 and GSDMD in the hippocampi of AD rats, which is consistent with the results of ELISA and Western blot. Use ImageJ software to measure the average optical density of A*β* in the hippocampal slices, and calculate the number of positive cells of NLRP3 and GSDMD in each field of view. The scale bar of A*β* is 200 um, the scale bar of NLRP3, and GSDMD is 100 um. Statistics and results display methods are the same as before.

Pyroptosis-related proteins are highly expressed in the brains of APP/PS1 transgenic mice [37]. A*β* triggers neuroinflammation by activating the pyroptosis pathway, causing neuronal damage, which may be an important mechanism of AD pathogenesis. Therefore, we assessed the expression of proteins related to the classical pyroptosis pathway in the rat hippocampus using Western blotting. Quantitative analysis revealed that A*β*_23–35_ hippocampal injection led to significant increases in NLRP3, GSDMD, pro-caspase-1, and caspase-1 P20 expression levels. Compared with donepezil, which slightly downregulated the expression of these proteins, JDYZF appeared to be more effective (Figure 4(a)). Notably, in the nonclassical pathway of pyroptosis, caspase-11 is directly activated by LPS to induce pyroptosis. Accordingly, we assessed the expression of the caspase-11 precursor and its cleaved products. Surprisingly, the results were consistent with those of the levels of proteins in the classical pathway (Figure 4(b)). This finding was further confirmed by the immunohistochemical staining of hippocampal samples from each group (Figure 5). These data suggest that JDYZF reduces nerve injury and improves cognitive function by downregulating the expression of proteins related to the pyroptosis pathway.

### 3.4. JDYZF Reduces the Levels of IL-1*β* and IL-18 in Multiple Tissues of AD Model Rats

**Figure 6:**
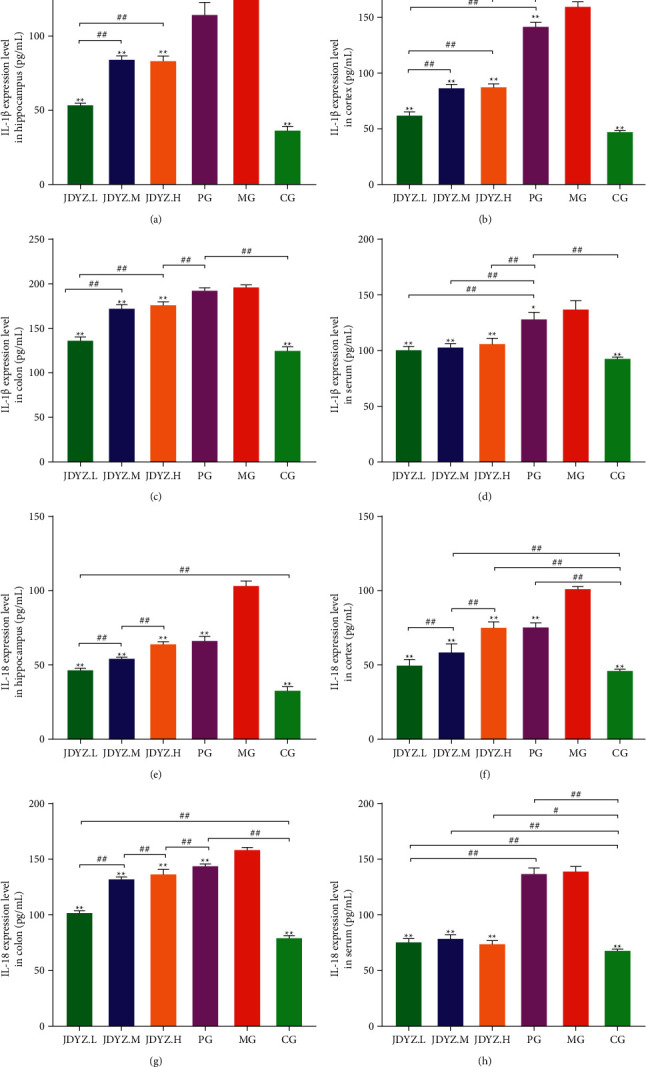
JDYZF can reduce the inflammatory response in AD rats by inhibiting the expression of IL-1*β* in the hippocampus (a), cerebral cortex (b), colon (c), and serum (d) and the expression of IL-18 in the hippocampus (e), cerebral cortex (f), colon (g), and serum (h).

Activated caspase-1 and caspase-11 cleave pro-IL-1*β* and pro-IL-18 and produce mature/activated IL-1*β* and IL-18, which aggravate local inflammation. We measured the levels of IL-1*β* and IL-18 in the hippocampus, cortex, colon, and serum samples of rats in each group. The levels of IL-1*β* and IL-18 were higher in the MG than in the CG. Donepezil and JDYZF decreased the levels of IL-1*β* and IL-18; however, the effect of JDYZF, especially JDYZ.L, was more apparent (Figures 6(a)–6(h)). This finding indicates that JDYZF may inhibit the inflammatory reaction in AD model rats.

### 3.5. JDYZF Has No Hepatotoxicity

**Figure 7:**
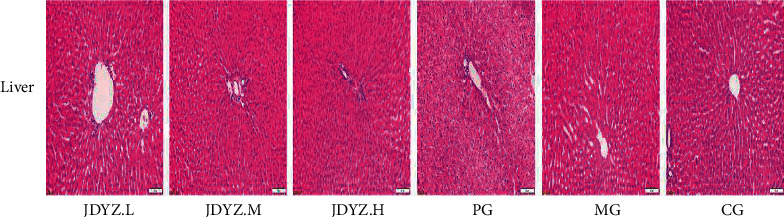
Representative images of HE staining of tissue sections from the rat liver. A*β* hippocampal injection and oral administration of JDYZF did not cause significant liver structural damage, indicating that JDYZF has no hepatotoxicity, while oral donepezil caused slight hepatic edema.

AD, as a chronic neurological disease, requires long-term oral drug treatment. Therefore, the safety of the drug is particularly important. We found that the administration of JDYZF did not cause significant changes in the liver structure based on the histological analyses of the livers of rats in each group (Figure 7). This finding indicates that oral JDYZF is safe and reliable for the treatment of AD.

## 4. Discussion

Currently, when the development of specific drugs to treat AD is blocked, various alternative methods are used. Clinical studies have shown that acupuncture at acupoints that replenish qi, resolve phlegm, and promote blood circulation can reduce Alzheimer's Disease Assessment Scale-cognitive subscale (ADAS-cog) and Clinician's Interview-Based Impression of Change-Plus (CIBIC-Plus) scores of AD patients [38]. In an animal experiment with similar acupoints, acupuncture reduced the protein expression levels of NLRP3, Caspase-1, and IL-1*β* in the hippocampi of AD model mice, inhibited the activation of microglia, and improved cognitive function [39]. Electroacupuncture at acupoints on the head has also been reported to improve the Montreal Cognitive Assessment (MoCA) scores of AD patients [40]. Animal experiments have also confirmed that electroacupuncture at acupoints on the head can enhance hippocampal and prefrontal cortex neuroprotection and regulate synaptic plasticity [41]. In addition, music therapy can alter the levels of neurotransmitters, autonomic nerve function, and neuronal connections to improve the memory and language ability of AD patients and reduce their mental symptoms [42, 43]. Aromatherapy can enhance neurogenesis in the limbic system of the brain through the projection of odour stimulation to improve cognitive impairment [11]. Evidence also supports the biological effects of pulsed electromagnetic fields in the treatment of AD, and their ability to improve cognitive impairment may be achieved by the modulation of insulin growth factors (IGFs) [12]. The emergence of these alternative therapies has enriched the treatment methods of AD and can be used in clinical treatment according to the specific conditions of the patient to maximize improvements in their clinical symptoms. In contrast, traditional Chinese medicine formulas are unique in the treatment of AD because of their multicomponent, multitargeted, and multipathway regulatory effects [14]. For example, the classic formula Shen-Zhi-Ling Oral Liquid, which is used for the treatment of AD, can reduce APP mRNA expression in the hippocampus, reduce amyloid deposition, reduce Caspase-3 expression, reduce neuronal apoptosis [44, 45], and increase hippocampal haemoglobin. The expression of oxygenase-1 (HO-1) and biliverdin reductase (BVR) can resist oxidative damage [46], regulate the insulin signal transduction pathway INR/PI3K/Akt, and improve glucose uptake, transport, and glycolysis in the brain [47]. The methods for improving AD are diversified and conform to the various pathological mechanisms of AD, thus achieving good clinical results.

A clinical study of JDYZF showed that it improved the Mini-Mental State Examination (MMSE) and MoCA scores in patients with cognitive impairment and had a positive effect on patients with AD [48]. In a previous mechanistic study, we found that the effect of *Coptis* polysaccharide, the extract of *Coptis chinensis* and the main component of JDYZF, on improving AD was multifaceted, which led us to hypothesize that JDYZF is more effective than other treatments.

The production and excessive accumulation of the toxin A*β* is an important factor driving the occurrence and progression of AD, and SPs are a pathological hallmark of AD [49]. SPs are formed by the accumulation of excess A*β*, which can induce the formation of NFTs, damage blood vessels, and induce neuronal loss [50]. Studies have shown that before numerous A*β* aggregates produce SPs, soluble A*β* oligomers cause nerve damage in many ways [51]. These oligomers can inhibit long-term potentiation (LTP) by enhancing the response of the N-methyl-D-aspartate receptor (NMDA) [52]. Oligomers can also directly interact with cell membranes to disrupt their permeability and bind with the cellular prion protein (PrP^c^) to disrupt the synaptic function of hippocampal neurons [53, 54]. Oligomers can bind with other disease-causing proteins associated with AD, such as TAR DNA-binding protein 43 (TDP-43), to form mixed oligomers and cause neuronal death [55]. Before the formation of SPs, neuroinflammation occurs in the AD brain [56], which may be related to the inflammatory response mediated by A*β* oligomers through receptors for advanced glycation end products (RAGEs), Toll-like receptors, and NLRs [57]. In this study, ELISA and immunohistochemical analyses confirmed that JDYZF reduced the expression level of soluble A*β*_1–42_ in the hippocampi of AD rats and reduced the A*β* deposition in plaques. This outcome may account for the mechanism by which JDYZF induces neuroprotective effects and reduces the neuroinflammatory response.

The response of NLRP3 to A*β* oligomers leads to the assembly of ASC and caspase-1 to form inflammasomes with NLRP3, which in turn activates GSDMD. Cell death and exogenous inflammatory factors are thereby induced via a process termed pyroptosis. During this process, ASC can bind to A*β* to further induce the production of A*β* oligomers [58], and inflammasomes can weaken the phagocytic function of microglia to take up A*β*, disrupting A*β* clearance [59]. The local inflammatory response caused by exogenous inflammatory factors can also promote the production and aggregation of A*β* [60], forming a vicious cycle between the occurrence of A*β* and pyroptosis and promoting the progression of AD. AD worsening can be slowed only by breaking this cycle. Studies have shown that knocking out the NLRP3 or Caspase-1 gene in APP/PS1 mice reduces the A*β* deposition in the brain and returns the LTP to nearly the baseline level [61]. Some researchers believe that the functional state of microglia in APP/PS1/NLRP3-/- or APP/PS1/Caspase-1-/- mice is shifted towards the M2 phenotype, which has anti-inflammatory effects and an enhanced ability to clear A*β*, which in turn has a neuroprotective effect [62]. Therefore, intervening in the NLRP3 inflammasome has become a new strategy for the treatment of AD. We herein showed that JDYZF reduced the protein expression levels of NLRP3, pro-caspase-1, and caspase-1 P20 in the hippocampi of AD rats and inhibited the excessive activation of NLRP3 inflammasomes, thereby inhibiting the maturation of GSDMD, breaking the vicious cycle between A*β* and pyroptosis and improving cognitive impairment in AD rats. In addition, studies have confirmed that activation of the NLRP3/caspase-1/GSDMD pathway damages the blood-brain barrier (BBB) [63]. When an imbalance in the intestinal flora in AD patients leads to an increase in intestinal LPS, BBB damage may lead to an increase in the LPS level in the brain [1, 64, 65]. Caspase-11 can directly recognize LPS and cleave GSDMD to initiate pyroptosis. Caspase-11 can also activate the NLRP3/caspase-1/GSDMD pathway, resulting in the expansion of pyroptosis [66], representing another vicious cycle that leads to the progression of AD. This study showed that JDYZF also reduced the expression of the caspase-11 precursor and its splicing body and interfered with the nonclassical pyroptosis pathway. Therefore, we hypothesize that JDYZF improves the intestinal flora imbalance in AD rats and reduces the levels of LPS in the intestine and brain. However, this hypothesis needs to be further explored in future studies.

IL-1*β*, located upstream of the inflammatory response in the brain, recruits peripheral immune cells to infiltrate the CNS and produce proinflammatory factors, induces APP expression to increase and promote the production and aggregation of A*β* [67–69], and plays an important role in the formation of a vicious cycle of A*β* and pyroptosis. In addition, the overexpression of IL-1*β* and IL-18 can damage the BBB, promote the expansion of central pyroptosis, and aggravate the inflammatory response [69]. Herein, JDYZF reduced the expression levels of IL-1*β* and IL-18 in multiple AD rat tissues, reduced the inflammatory response, and weakened the effect of inflammatory factors on the vicious cycle of A*β* production and pyroptosis.

## 5. Conclusion

In summary, the pathogenesis of AD is complicated and unclear. Clinicians should choose comprehensive treatment methods that are suitable for patients to improve their condition to the greatest extent. JDYZF has achieved good results as a clinical treatment for AD. It may reduce the expression and deposition of A*β*, inhibit the expression levels of pyroptosis-related proteins, reduce the neuroinflammatory response, and block the vicious cycles between A*β* and pyroptosis, pyroptosis and LPS, and inflammation, A*β* and pyroptosis, thereby improving cognitive impairment in AD rats. By exploring AD treatment mechanisms, we hope to provide an alternative treatment.

## Data Availability

The data used to support the results of this study are included in the article.
